# Global DNA methylation and cellular 5-methylcytosine and H4 acetylated patterns in primary and secondary dormant seeds of *Capsella bursa-pastoris* (L.) Medik. (shepherd’s purse)

**DOI:** 10.1007/s00709-021-01678-2

**Published:** 2021-07-02

**Authors:** Sara Gomez-Cabellos, Peter E. Toorop, María Jesús Cañal, Pietro P. M. Iannetta, Eduardo Fernández-Pascual, Hugh W. Pritchard, Anne M. Visscher

**Affiliations:** 1 Department of Comparative Plant and Fungal Biology, Royal Botanic Gardens, Kew, Wakehurst Place, Ardingly, RH17 6TN West Sussex UK; 2 Departamento de Biología de Organismos y Sistemas, Universidad de Oviedo, C/Catedrático Rodrigo Uría, 33006 Oviedo, Spain; 3 The James Hutton Institute, Invergowrie, Dundee, DD2 5DA Scotland UK

**Keywords:** Primary seed dormancy, Secondary seed dormancy, Global DNA methylation, Immunolocalization, Histone acetylation dynamics, DNA methylation dynamics

## Abstract

Despite the importance of dormancy and dormancy cycling for plants’ fitness and life cycle phenology, a comprehensive characterization of the global and cellular epigenetic patterns across space and time in different seed dormancy states is lacking. Using *Capsella bursa-pastoris* (L.) Medik. (shepherd’s purse) seeds with primary and secondary dormancy, we investigated the dynamics of global genomic DNA methylation and explored the spatio-temporal distribution of 5-methylcytosine (5-mC) and histone H4 acetylated (H4Ac) epigenetic marks. Seeds were imbibed at 30 °C in a light regime to maintain primary dormancy, or in darkness to induce secondary dormancy. An ELISA-based method was used to quantify DNA methylation, in relation to total genomic cytosines. Immunolocalization of 5-mC and H4Ac within whole seeds (i.e., including testa) was assessed with reference to embryo anatomy. Global DNA methylation levels were highest in prolonged (14 days) imbibed primary dormant seeds, with more 5-mC marked nuclei present only in specific parts of the seed (e.g., SAM and cotyledons). In secondary dormant seeds, global methylation levels and 5-mC signal where higher at 3 and 7 days than 1 or 14 days. With respect to acetylation, seeds had fewer H4Ac marked nuclei (e.g., SAM) in deeper dormant states, for both types of dormancy. However, the RAM still showed signal after 14 days of imbibition under dormancy-inducing conditions, suggesting a central role for the radicle/RAM in the response to perceived ambient changes and the adjustment of the seed dormancy state. Thus, we show that seed dormancy involves extensive cellular remodeling of DNA methylation and H4 acetylation.

**Supplementary Information:**

The online version contains supplementary material available at 10.1007/s00709-021-01678-2.

## Introduction

Seed dispersal enables a plant’s genetic material to travel through space and time, leading to the establishment of a new plant in a suitable environment. The time between seed dispersal and completion of germination can be short or long; thus, seeds have evolved a series of dormancy strategies to survive this interval (Footitt and Finch-Savage 2017). These strategies prevent responses to short-lived, out of season environmental changes. Moreover, seeds of some species can remain dormant in the soil for many years until the conditions are suitable for the resulting plant to survive (Footitt et al. 2013).

Seed dormancy is a complex trait that is defined as a barrier to the completion of germination of an intact viable seed under normally favorable conditions (Hilhorst 1995; Bewley 1997; Baskin and Baskin 2004). In contrast, a completely non-dormant seed has the capacity to germinate over the widest range of environmental conditions possible for that genotype (Baskin and Baskin 2004). In a population of seeds in the soil seed bank, individual seeds can have different levels of dormancy (Footitt and Finch-Savage 2017).

Primary dormancy is the innate dormancy possessed by seeds when they are dispersed from the mother plant (Benech-Arnold et al. 2000), the level depending on the environment during seed development, and the physiological characteristics imposed by the mother plant (Bewley and Black 1994). Secondary dormancy occurs post-dispersal and after primary dormancy has been released. Entrance into secondary dormancy can be induced in seeds with non-deep physiological dormancy, if the right set of external signals to germinate are incomplete (Hilhorst 1998). Secondary dormancy may be relieved and non-dormant seeds may complete germination if the right conditions are encountered. Induction and relief of secondary dormancy can occur during successive seasons, leading to an annual dormancy cycle in the seed bank (Hilhorst 1998).

*Capsella bursa-pastoris* (L.) Medik. seeds display primary dormancy and are able to enter secondary dormancy and undergo seasonal dormancy cycles in the soil (Toorop et al. 2012). Although secondary dormancy is of great importance in germination ecophysiology, little is known about this phenomenon in *Capsella* (Neuffer and Hurka 1986). Shepherd’s purse has a worldwide distribution, with the exception of the extremely dry tropical environments (Neuffer and Eschner 1995; Hurka and Neuffer 1997), and it has become one of the five most widely distributed flowering plants on the planet (Hintz et al. 2006). Moreover, it has a very small phylogenetic distance to the academic model species *Arabidopsis thaliana*, which, contrary to shepherd’s purse, seems to be uncompetitive and rare in the wild (Hintz et al. 2006). Therefore, *C. bursa-pastoris* is an ideal model in which to study these phenomena.

In both *A. thaliana* (Donohue et al. 2007) and *C. bursa-pastoris* (Toorop et al. 2012) seeds, imbibition at high temperatures under darkness followed by exposure to light has been shown to be an effective means to studying differences in secondary dormancy between genotypes. Secondary dormancy induction by high temperatures stimulates the upregulation of ABA-mediated dormancy pathways and of genes involved in GA catabolism (Martel et al. 2018).

Gene expression is influenced by chromatin structure, which is controlled by processes often associated with epigenetic regulation (Chinnusamy and Zhu 2009). Whilst global acetylation of histones is linked to the “open” configuration of chromatin named euchromatin, global DNA methylation is linked to the “closed” configuration named heterochromatin. Interaction between DNA methylation and histone modifications to jointly control gene expression in plants, fungi, or mammals has been demonstrated (Fuks 2005).

Post-translational histone modifications confer different physical properties to chromatin, altering the accessibility of transcription machinery to the DNA (Itabashi et al. 2018). Whilst acetylation of a histone lysine residue activates gene expression by neutralizing the positive charge of lysine and reducing the interaction between histones and DNA (Lee and Workman 2007), methylation of histones is more complex as it is associated with both activation and repression of transcription (Mathieu et al. 2005; Chen et al. 2010). DNA methylation consists of the addition of a methyl group onto the C5 position of cytosine to form 5-methylcytosine (5-mC). It is required for maintaining genomic structure and stability, regulating gene expression, imprinting, growth, development (An et al. 2017), in addition to silencing transposable elements (TEs), repetitive sequences, and transgenes (Bartels et al. 2018). The effects of this epigenetic mark on gene transcription relate to their differential distribution in the genes, being associated with repression in gene promoters and with activation in gene bodies, depending on the level of methylation (Zilberman et al. 2007).

The role of epigenetic processes in the regulation of dormancy was first elucidated in potato meristems during dormancy progression, particularly changes in 5-mC and histone H3 and H4 multi-acetylation (Law and Suttle 2002, 2004). Moreover, different expression patterns of histone acetyltransferases and deacetylases were found between dormant and non-dormant seeds of *A. thaliana* (Cadman et al. 2006). Low dormancy mutants revealed that *HISTONE UBIQUITINATION1* (*HUB1*) was required for the mono-ubiquitination of H2B, which in itself is necessary for H3K4me3 and H3K79me3 histone modifications (Liu et al. 2007). Not only did the spatial distribution of histone H3 and H4 acetylation change in *Brachypodium distachyon* embryos during the four stages of seed development (maturation, desiccation, imbibition, germination) but so did DNA methylation (Wolny et al. 2017). However, embryos in a dormant state were not studied.

Whilst the role of epigenetics in the control of gene expression is well established, a comprehensive characterization of the levels of global DNA methylation and histone H4 acetylation of seeds, or the distribution of these epigenetic marks in space and time in different states of dormancy, had not been undertaken previously. We hypothesized that global DNA methylation and global H4 acetylation had a dynamic role during the induction and maintenance of seed dormancy in shepherd’s purse, adjusting the seeds’ dormancy state through regulation of dormancy controlling genes in response to environmental signals. In our hypothesis, 5-mC levels should increase in relation to the depth of dormancy over time and H4 acetylation levels decrease during this process. In addition, these epigenetic changes would be reflected especially in the meristems.

## Material and methods

### Plant material

The capacity for induction of secondary seed dormancy in *C. bursa-pastoris* seeds was analyzed in nine accessions (SCRI -156, -177, -367, -416, -469, -707, -773, -799, and -937 from the Scottish Crop Research Institute). These were derived originally from soil samples taken from 34 fields around the Farm Scale Evaluation sites in 2001 in the UK (Champion et al. 2003). Plants were then grown from these seeds over the summer/autumn of 2005 and 52 genotypes were classified based on previous knowledge of their flowering times (Iannetta et al. 2007). Seeds from the accessions studied were stored at 15 °C with 15% of RH in the Millennium Seed Bank, Wakehurst Place, Royal Botanic Gardens, Kew, UK.

Seeds from accession SCRI -773 of shepherd’s purse were sown and grown in Oviedo, Asturias, Spain, from November 2013 to March 2014, to produce primary dormant seeds. Seeds were surface sterilized in 1 mL of sodium hypochlorite (NaOCl) with 0.01% (v/v) Tween20 for 5 min with shaking. After washing three times with sterile distilled water, seeds were germinated in Petri dishes on two layers of filter paper Whatman no. 1 that were moistened with sterilized distilled water. Dishes were placed at 30 °C and the seeds exposed to cycles of light/dark of 12 h each, with light provided by white fluorescence tubes (radiometric flux density of 50–100 W m^−2^). Once germinated, seeds were transferred to 2.5 cm^2^ plastic modules in a greenhouse in Oviedo, Asturias, Spain. When seedlings were well established, they were transferred to medium size peat pots for 6 weeks and then to large size peat pots for the rest of their life cycle. These plants were considered replicate plants of the genotype. Plants were bagged with paper bags individually when siliques showed the first signs of dehiscence and left to dry without watering in a warm and dry location.

Seeds were harvested from individual plants and then mixed to form 8 different pools that were stored at 4 °C in hermetic glass containers with dried silica gel and a RH and temperature measuring dispositive for the rest of the project. Germination tests were carried out throughout that period to corroborate changes in dormancy state (data not shown). Three conditions were used: (1) seeds imbibed in water at 30 °C with a 12 h-photoperiod as an optimum test for non-dormant seeds; (2) seeds imbibed in water but exposed to alternate cycles of light/dark (12 h) and high/low temperatures (25/10 °C), as this treatment is known to partially break primary dormancy; (3) seeds imbibed in 10 mM KNO_3_ with alternate cycles of light/dark (12 h) and high/low temperatures (25/10 °C), as a way of completely breaking primary dormancy. For the subsequent experiments, seeds from the largest three pools (1, 3 and 5) were used as biological replicates.

### Germination tests

The selection of accessions with contrasting secondary dormancy depth was based on germination tests under dormancy-inducing conditions. The experiments comprised nine seed accessions (SCRI -156, -177, 367, -416, -469, -707, -773, -799 and, -937). All seed lots had been stored at 15 °C and 15% RH since 2006, losing any residual primary dormancy by after-ripening as corroborated by germination tests at 30 °C (described above).

To test seed lot capacity for secondary dormancy induction, seeds of all genotypes were imbibed in water and incubated in darkness by wrapping Petri dishes with three layers of aluminum foil for different periods of time (0 (directly in light conditions), 1, 2, 3, 5, 7, or 14 days) at a constant temperature of 30 °C. For each experiment, three replicates of approximately 50 seeds per treatment (each replicate corresponding to a different mother plant of the same accession) were placed in separate 50 mm Petri dishes on two layers of Whatman no. 1 filter paper soaked with 1.5 mL of water.

Germination in darkness was scored at the end of each dark-incubation period (1 to 14 days), and the seeds transferred to a 12-h photoperiod at the same temperature (30 °C). Germination was scored twice daily under a binocular microscope. Seeds with a protruding radicle > 1 mm were considered to have germinated. To exclude loss of viability as a possible cause of any differences in germination level, non-germinated seeds were left to dry for 7 days (4 days in the laboratory; and 3 days in a room at 15 °C and 15% RH). Thereafter, seeds were soaked in 1.5 mL of a 10 mM KNO_3_ solution and placed at 25/10 °C with a 12-h photoperiod coinciding with the warm temperature phase. Seeds that had not previously germinated but did under these conditions were scored as viable and previously dormant; those that did not germinate, as inviable. All experiments used controlled environments as specified above.

Seed germination was quantified as final germination proportion. A fully factorial generalized linear model (GLM) was fitted to the final germination proportions, with logit link and binomial distribution (accession or darkness as fixed factors, plus their interaction) using R (RC Team 2013).

### DNA extraction

For DNA extraction from *C. bursa-pastoris* seeds exposed to conditions listed in Supplementary table ST. 1, a custom extraction protocol developed by our research group was applied. We used the commercial extraction buffer AP1 (DNeasy® Plant Mini Kit, Qiagen), along with disposable chromatographic mini spin columns with an anion exchange matrix. After lysis and a pre-purification step, the lysate was mixed with a chaotropic salt that allowed DNA to bind to the anion exchange matrix. Following this, DNA was purified using washing steps and eluted from the anion exchange column by adding sterilized milliQ water. Times and procedures were optimized for shepherd’s purse seed material. Fifty milligrams of dry or imbibed seeds were used as starting material for each sample. Samples were frozen and ground in liquid nitrogen with mortar and pestle under liquid nitrogen immediately before the procedures.

The homogenized material was incubated with 1 mL of the extraction buffer AP1 (Qiagen), combined with 20% (v/v) SDS solution (490 µL), 5% (v/v) β-mercaptoethanol, and 1% (v/v) RNase A (added just prior use), at 65 °C for 1 h. The solution was cooled down and potassium acetate (3 M C_2_H_3_KO_2_) added (1:3 volume) and mixed by vortex. The resulting solution was placed on ice for 20 min. Centrifugation was carried out at 21,000* g* for 5 min at 4 °C and the pellet was discarded. The lysate was transferred to a new collection tube and another precipitation step with potassium acetate was performed. The pellet was discarded, and the lysate was transferred to a silica mini spin column for DNA purification (Econospin® mini spin column) and centrifuged at 18,000* g* for 2 min at RT. One and a half volumes of Guanidine-HCl (0.66 M CH_5_N_3_-HCl in 63% (v/v) ethanol) were then added to the flow through and mixed in by pipetting. The solution was transferred to a silica spin mini column and centrifuged at 11,000* g* for 1 min. The flow through was discarded and the column was transferred to a new collection tube. Five hundred µL of washing solution (0.025 M NaCl, 0.005 M Tris–HCl (pH 7.5), and 0.05 mM EDTA in 75% (v/v) ethanol absolute) were added to the column and this was then centrifuged at 11,000* g* for 1 min. The flow through was discarded and another 500 µL of washing solution were added and the column centrifuged at 18,000* g* for 3 min before the flow through was discarded again. The column was transferred to a new collection tube to which 30 µL of sterilized milliQ water at 70 °C were added and incubated for 5 min. The column on the collection tube was centrifuged at 11,000* g* for 1 min at room temperature (RT) and the eluted DNA was kept on ice. Addition of water, centrifugation and collection of the eluted DNA were repeated twice.

Genomic DNA quantity and quality was evaluated by agarose gel electrophoresis and by measuring the 260/280 and the 260/230 absorbance ratios (A260/280 and A260/230, respectively) using a NanoDrop™ Spectrophotometer (ThermoScientific™). DNA was subjected to gel electrophoresis in 1% agarose gels, stained with ethidium bromide, and visualized using a UV transilluminator. DNA was extracted for the conditions indicated in Supplementary table ST. 1.

### Quantification of global DNA methylation

MethylFlash™ Global DNA Methylation (5-mC) ELISA Easy Kit (Colorimetric) (Epigentek, NY) was used according to the manufacturer’s instructions. One hundred nanograms of purified DNA from each sample were added to an ELISA plate where the methylated fraction of DNA was detected using capturing and detecting antibodies. Optical density (OD) intensity at 450 nm was read with a microplate spectrophotometer and this was proportional to the amount of methylated DNA. For quantification of global DNA methylation levels, a standard curve was generated using methylated DNA standards provided in the kit. The value for each sample was calculated as a ratio of the OD of the sample relative to the OD of the standard, after subtracting the negative control readings (Supplementary figure SF. 1). The assay was performed in duplicate per biological replicate, meaning that per accession and condition, six samples were analyzed.

Data were assessed for significance (*P* < 0.05) by one-way ANOVA (dormancy type as factor) and two-way ANOVA analyses (dormancy type and duration as main factors) using R software v3.3.0. The ANOVA analyses were combined with Tukey’s HSD (honest significance difference) test for post hoc comparisons of means using “Agricolae” v.1.3–1 (Mendiburu 2019) package in R.

### Scanning electron microscopy, fixation, and processing

After 1 day of imbibition, testae were removed from at least three *C. bursa-pastoris* seeds, and from each whole mature embryo, one cotyledon was dissected to expose the shoot apex (epicotyl). The material was fixed overnight at 4 °C under a vacuum in 2.5% (v/v) glutaraldehyde and 4% (w/v) paraformaldehyde in 0.2 M cacodylate buffer (pH 7.2). Fixed embryos were washed three times in phosphate buffer saline (PBS) and stored in the fixative at 4 °C until use. Just prior to use, samples were washed in cacodylate buffer and post-fixated in 1% (w/v) osmium tetroxide (OsO_4_) cacodylate buffer for 1 h. The material was then dehydrated in an alcohol series and washed twice with acetone. The samples were critical-point dried in liquid CO_2_ and sputter-coated with gold/palladium. Scanning electron microscopy was performed with a JSM 7400F field emission scanning electron microscope at an accelerating voltage of 5 kV.

### Immunohistochemical detection of 5-mC and H4Ac

Seeds from accession SCRI -773, mother plant 6, were used as non-dormant seed material. At least three replicate samples per treatment were studied. After each of the treatments indicated in Table 1, whole seeds were fixed in 4% paraformaldehyde in PBS (w/v) overnight at 4 °C under a vacuum. To facilitate penetration of the fixative, seed testae were punctured with a needle under safe green light conditions before fixation.

**Table 1:** Immunolocalization conditions studied

Primary dormancy conditions -773	Secondary dormancy conditions -773
Dry seeds	Dry seeds
1 d 30 °C 12 h light	1 d 30 °C 12 h light
14 d 30 °C 12 h light	1 d 30 °C dark
	3 d 30 °C dark
	7 d 30 °C dark
	14 d 30 °C dark

Fixed seeds were washed three times in PBS and stored in 0.1% paraformaldehyde in PBS at 4 °C until use. Just prior to use, samples were washed in PBS, embedded in Tissue-Tek compound and frozen for sectioning. Once frozen, seeds were sectioned at 40–50 µm thickness using a Leica CM1510-S cryomicrotome (Leica Instruments), mounted on coated microscope slides, air-dried, and stored at − 20 °C until use for immunohistochemistry.

After equilibration to RT, sections were washed three times in PBS, dehydrated in an ascending ethanol series (25%, 50%, 75%, and 100%; 5 min each) and subsequently rehydrated in a descending ethanol series (100%, 75%, 50%, and 25%; 5 min each). After a washing step with PBS, samples were subjected to an enzymatic digestion of cell walls by incubation in 2% (w/v) Cellulase Onozuka R-10 and 2% (w/v) Macerozyime R-10 (©Duchefa Biochemie) in PBS for 1.5 h at 30 °C followed by 45 min in 0.1% Tween 20 in PBS (v/v) at RT. DNA was denatured for 5-mC detection with 2 N HCl for 30 min and incubated with 5% of bovine serum albumin (BSA) in PBS (w/v) and 0.1% Triton (v/v) for 30 min to prevent unspecific binding.

Sections were incubated for 2 h with the mouse monoclonal anti-5mC antibody (Diagenode, Cat. N. C15200081, Liege, Belgium) or with the rabbit polyclonal anti-H4Ac antibody (Merk KGaA, Cat. N. 06–866, Darmstadt, Germany) diluted 1:50 in 1% BSA in PBS (w/v) with 0.1% Triton X-100 (v/v). After two rinsing steps in 0.1% Tween 20 in PBS (v/v), sections were incubated for 1 h in darkness with either the Alexa Fluor® 488-labeled anti-mouse polyclonal secondary antibody (Thermo Fisher Scientific Inc., Cat. N. A11001) for 5-mC detection or the Alexa Fluor® 488-labeled anti-rabbit polyclonal secondary antibody (Thermo Fisher Scientific Inc, Cat. N. A11008) for H4Ac detection. Secondary antibodies were diluted 1:25 in 1% BSA in PBS (w/v) with 0.1% Triton X-100 (v/v). Nuclei were counterstained with a 4′,6-diamidino-2-phenylindole (DAPI) solution (1 µg mL^−1^, 0.1% Triton in PBS (v/v)) for 30 min in darkness, washed in distilled water, and mounted in Mowiol® 4–88 (Sigma-Aldrich).

A control was included for all section types and materials used by performing the whole immunohistochemical protocol with substitution of primary antibodies by PBS. Controls showed no fluorescence signal in the nuclei.

### Image analysis

Fluorescence was examined under a confocal microscope (Leica Microsystems TCS-SP8-AOBS). Confocal optical sections were collected at 0.5 µm z-intervals. Images of maximum projections from each z-series were obtained with the open source image processing software ImageJ (version 1.52i) (Rasband, W.S., National Institutes of Health, Bethesda, MD, USA, http://imagej.nih.gov/ij). For confocal series capture, the same settings (pinhole size, gain, offset, and laser intensity) were used in all the experiments performed using the same primary antibody (anti-5-mC or anti-acetylated histone H4). We used visual assessment (performed by three authors) of one good quality image per treatment to compare differences in fluorescence (number of nuclei and intensity) between areas of the seeds (Supplementary figure SF. 8) and across treatments, a procedure that has been applied by other authors such as Meijón et al. (2009) or Santamaría et al. (2009).

In addition, the number of nuclei with fluorescence and the fluorescence intensities of those nuclei were also quantitatively measured for one image per treatment (whole embryo). The contour of each nucleus was manually outlined and the average fluorescence intensity per area was obtained. This is the sum of the fluorescence on each pixel within the outlined area (a number from 0 to 255 per pixel) divided by the total number of pixels of this area. Average fluorescence intensities were quantified using ImageJ (version 1.52i) image processing software. Data are presented as total number of nuclei and mean intensity value for each treatment.

DNA methylation and histone acetylation results are reported as mean ± SE. Differences among treatments means were calculated by one-way ANOVA with post hoc Tukey’s HSD with *P* = 0.05 using R software (version 3.5.2). All statistical tests were performed using the R packages: ggplot2 v.3.2.0 (Wickham 2016) and Agricolae v.1.3–1 for Tukey’s post hoc test (Mendiburu 2019).

## Results

### Germination tests

**Fig. 1:**
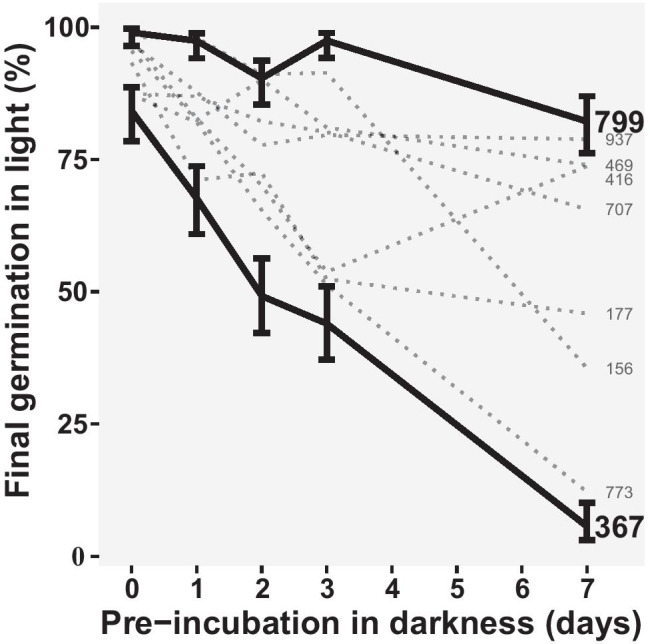
Differential rate of dark-induced dormancy induction among accessions (SCRI -156, -177, -367, -416, -469, -707, -773, -799, and -937) of *C. bursa-pastoris* in water and times of incubation in darkness tested (0, 1, 2, 3, and 7 days). Brackets represent the 95% binomial confidence interval

Germination tests were performed to identify shepherd’s purse accessions naturally differing in secondary seed dormancy depth. Out of nine accessions tested, -367 and -799 had the most extreme responses to secondary seed dormancy induction (Fig. 1). Comparing these two, the differences in the final seed germination after dark-induction were significant (*P* < 0.05; main effect’s Χ^2^ = 291.2, *P* < 0.001). Accession -367 showed less germination than accession -799 at all time intervals (main effect’s Χ^2^ = 89.8, *P* < 0.001), and it also responded more to dormancy induction (interaction’s Χ^2^ = 19.5, *P* < 0.001), with the rate of secondary dormancy induction approximating to a fall of 11% in germination per day in -367, compared with 2% per day for -799. This indicates that accession -367 has about a five-time higher propensity to enter secondary dormancy (Supplementary table ST. 2). The other seven seed accessions had intermediate sensitivities to secondary dormancy induction.

Seeds from three different mother plants per accession (-367.1, -367.2, -367.3, as well as -799.1, -799.2, and -799.4) were selected as biological replicates for the subsequent methylation and acetylation studies (in addition to the primary dormant seeds described in the “Material and methods” section).

### Quantification of global DNA methylation

Global DNA methylation was analyzed with the MethylFlash™ Global DNA Methylation (5-mC) ELISA Easy Kit (Colorimetric), which includes a standard curve with a range for quantification of methylation from 0.1 to 5.0%. (Supplementary figure SF. 2). The lowest DNA methylation level measured in the seed lots was 1.63%, and the highest 12.05%. All the measurements higher than 5.0% can therefore only be compared within this assay.

**Fig. 2:**
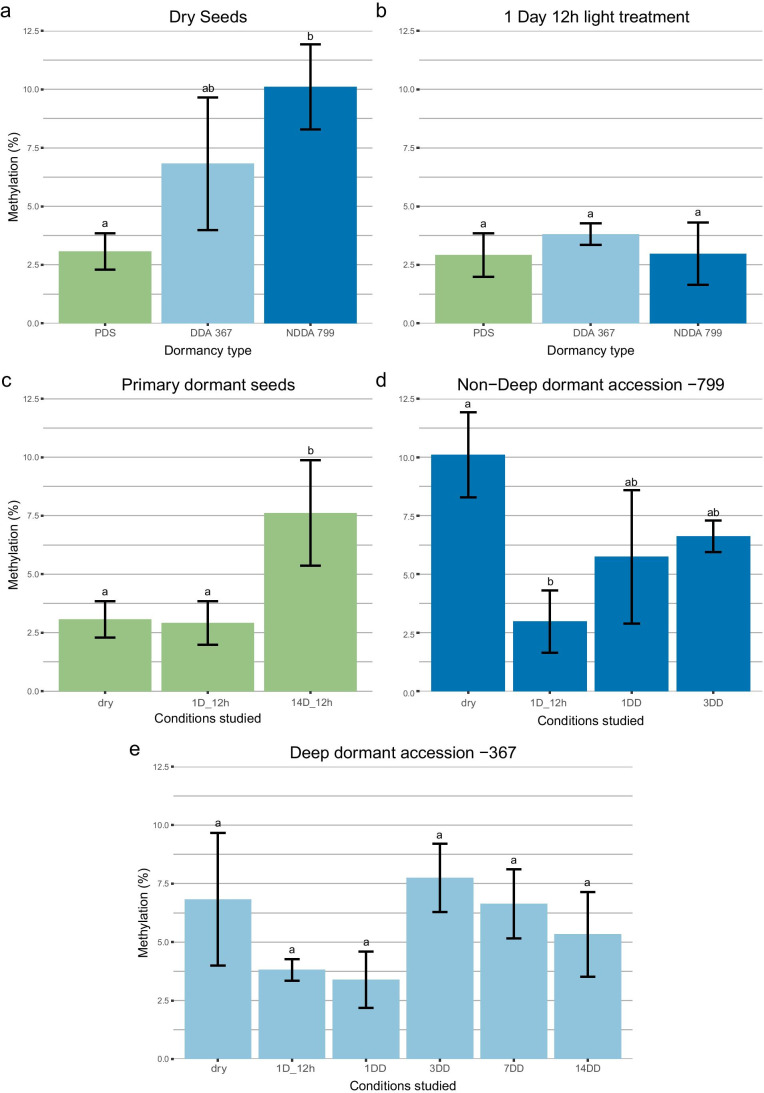
Histogram representing the values of 5-mC percentages of total cytosines. **a** Primary dormant dry seeds, dry seeds from the deep dormant accession -367 and dry seeds from the non-deep dormant accession -799. **b** Primary dormant seeds, non-dormant seeds from the deep dormant accession -367, and non-dormant seeds from the non-deep dormant accession -799 imbibed in water for 1 day with 12 h of light. **c** All the conditions studied for primary dormant seeds. **d** All the conditions studied for the non-deep dormant accession -799. **e** All the conditions studied for the deep dormant accession -367. Statistical analysis of global DNA methylation percentage means for every three biological replicates per physiological condition with two technical replicates. Different letters indicate significant differences at *P* < 0.05 (Tukey’s HSD test). Bars represent standard deviation. PDS, primary dormant seeds; DDA 367, deep dormant accession -367; NDDA 799, non-deep dormant accession -799; DD, days in darkness

DNA methylation levels in seeds from the deep and the non-deep dormant accessions (-367 and -799, respectively) revealed similar patterns across time (Fig. 2). The ANOVA statistical analysis showed that there was a significant interaction between the duration of the treatment to which seeds were exposed and the levels of global DNA methylation (*P* < 0.001), in the three types of dormancy studied (primary dormancy, deep and non-deep secondary dormancy).

Global DNA methylation levels were significantly different (*P* < 0.01) between primary dormant dry seeds (3.07 ± 0.82%) and non-dormant dry seeds from the non-deep dormant accession -799 (10.11 ± 1.91%). At the same time, non-dormant dry seeds from the deep dormant accession -367 had lower global DNA methylation levels (6.83 ± 3.15%) than the non-dormant dry seeds from the non-deep dormant accession -799, although the difference was not significant (Fig. 2a).

Global DNA methylation levels of seeds imbibed in water for 1 day with a 12-h photoperiod showed the lowest levels of DNA methylation throughout all the treatments studied. These levels did not differ significantly between the three types of seed dormancy states studied (Fig. 2b).

With respect to primary dormant seeds, global DNA methylation levels of seeds imbibed in water for 1 day with a 12-h photoperiod (2.91 ± 1.02%) did not differ significantly from those of primary dormant dry seeds (3.07 ± 0.83%). However, when these seeds were imbibed for a longer period of 14 days under the same conditions, DNA methylation levels increased significantly compared to only 1 day (7.61 ± 2.26%) (*P* < 0.05; Fig. 2c).

Global DNA methylation levels of seeds from the deep dormant accession -367 did not differ significantly throughout all the treatments. However, seeds imbibed in water for 1 day with or without a 12-h photoperiod presented lower levels than deep dormant dry seeds and very similar levels between them. In the induction of secondary dormancy of the deep dormant accession, seeds imbibed in water for 3 days in darkness had the highest global DNA methylation, with increasingly lower levels of DNA methylation after 7 and 14 days in darkness, although higher levels than seeds for 1 day in darkness.

Dry seeds from the non-deep dormant accession -799 had significant differences from non-deep dormant seeds imbibed for 1 day in water (12-h photoperiod) (*P* < 0.001). Non-deep dormant seeds imbibed in darkness for 1 or 3 days did not differ significantly from dry seeds or seeds imbibed in water for 1 day (12-h photoperiod), although their methylation levels were higher than the latter ones.

### Scanning electron microscopy

**Fig. 3:**
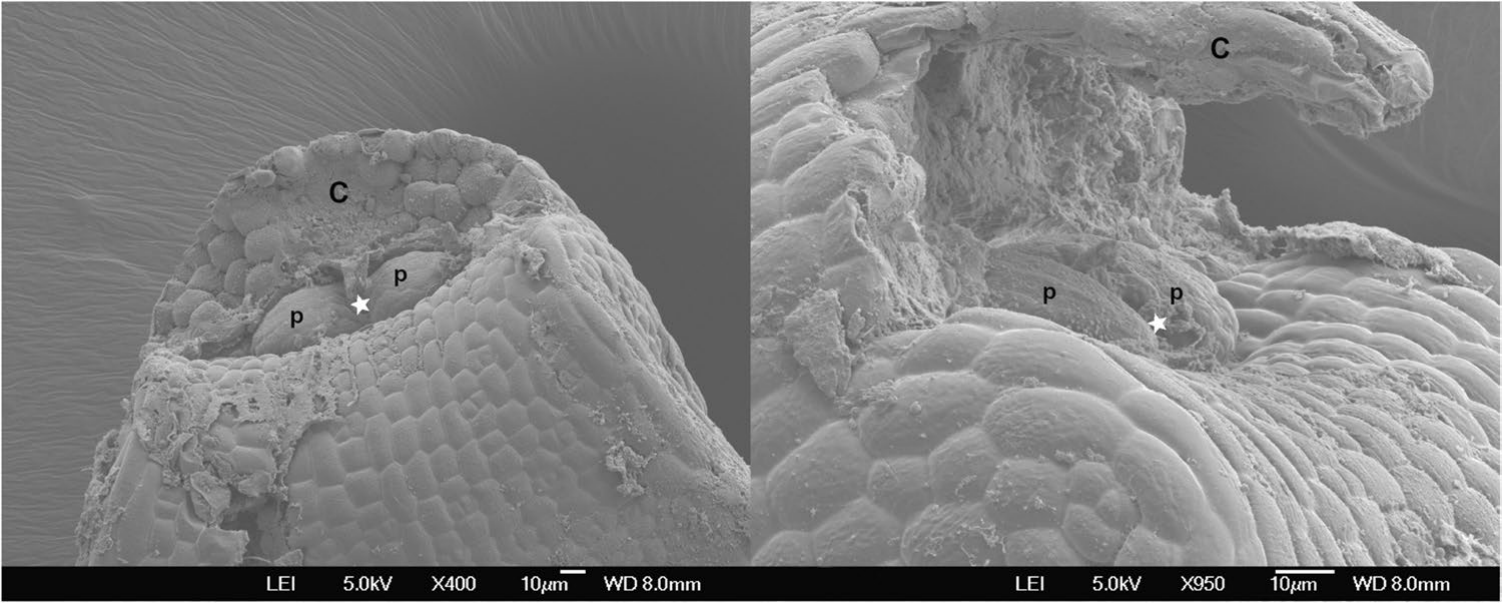
SEM of mature embryo apices of shepherd’s purse imbibed in water for 1 day where one cotyledon has been removed (**C**). First true leaf primordia (**p**) and SAM (white star) are indicated. Scale bars of 10 µm

SEM images showed that the shoot apical meristem of shepherd’s purse mature embryos is flanked by two leaf primordia (Fig. 3) that are between 35–40 µm wide and 20 µm long.

### Immunohistochemical detection of 5-mC and H4Ac

#### Primary dormant seeds

##### DNA methylation

**Fig. 4:**
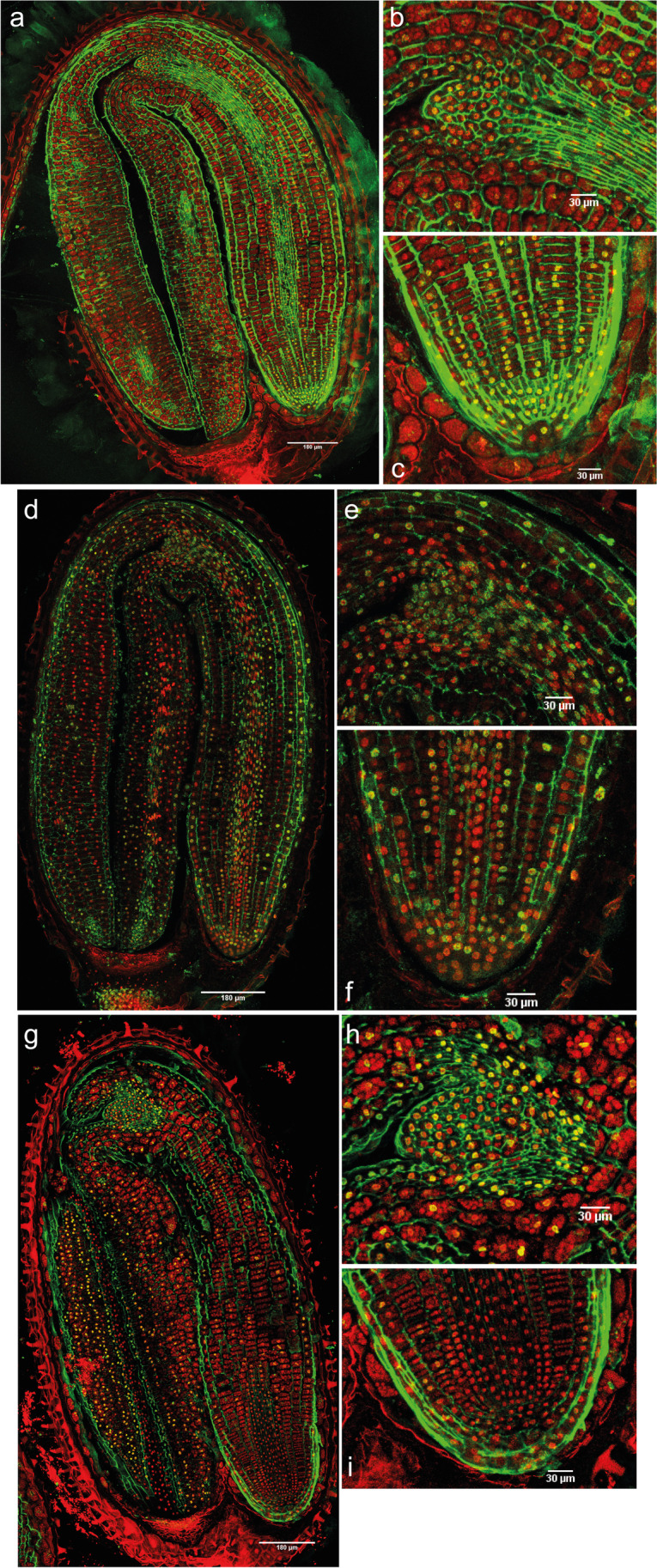
Merged images of immunodetection of 5-mC using confocal microscopy in primary dormant seeds. **a** Primary dormant dry seeds with (**b**) detail of the shoot apex and (**c**) detail of the root apex. **d** Primary dormant seeds imbibed in water for 1 day with 12 h of light with (**e**) detail of the shoot apex and (**f**) detail of the root apex. **g** and **h** Primary dormant seeds imbibed in water for 14 days (12-h photoperiod) with (**i**) detail of the shoot apex and (**j**) detail of the root apex. DAPI (in red, false color) and Alexa 488, immunostaining 5-mC (in green). Scale bars of 180 and 30 µm

The intensity of the 5-mC signal in primary dormant dry seeds was detected in different areas, although the intensity of the signal was low (Fig. 4a). In the shoot apex, even though the leaf primordia had 5-mC, immunofluorescence was not detected in the SAM (Fig. 4b). On the other hand, in the radicle, especially in the RAM, the immunosignal was the most intense (Fig. 4c). After 1 day of imbibition with 12 h of light, there was an increase in the signal intensity (Fig. 4d–e, Supplementary figure SF. 4) and the embryo had an increase in the number of marked nuclei in comparison to dry seeds (Supplementary table ST. 3). Standing out was the shoot apex, where the SAM had immunosignal (Fig. 4e).

With an imbibition time of 14 days with light, the immunofluorescence signal in the hypocotyl, elongation zone, and radicle disappeared, compared to seeds imbibed for 1 day (Fig. 4g, i). On the other hand, there was an increase in the number of marked nuclei in the SAM, vascular tissue of the shoot apex, and in the cotyledons (Fig. 4h). Overall, the intensity per nucleus and total intensity (nuclei × mean intensity) was highest in the whole embryo at 14 days (Supplementary figure SF. 4, Supplementary table ST. 3).

##### Histone H4 acetylation

**Fig. 5:**
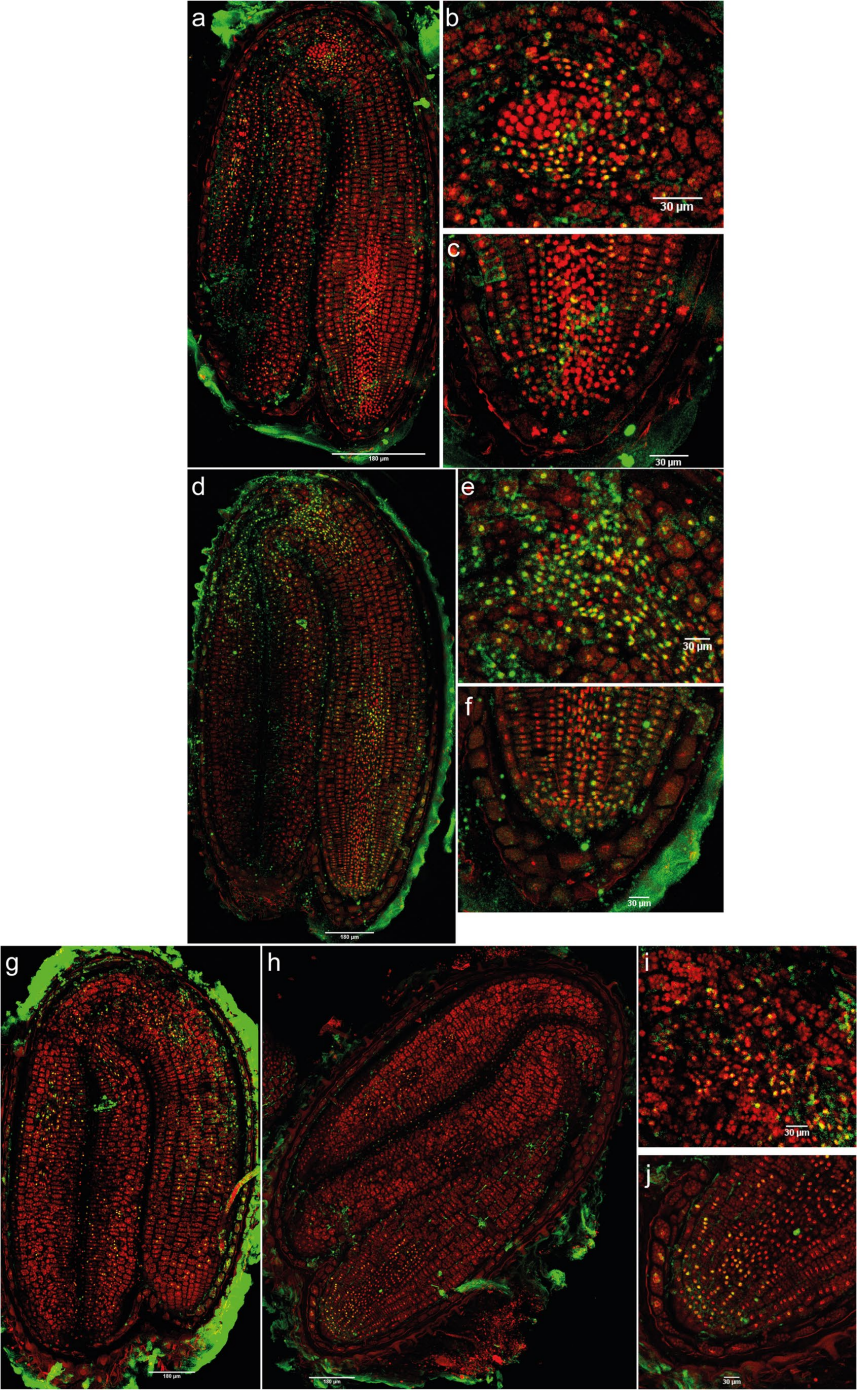
Merged images of immunodetection of H4Ac using confocal microscopy in primary dormant seeds. Longitudinal sections. **a** Primary dormant dry seeds with (**b**) detail of the shoot apex and (**c**) detail of the root apex. **d** Primary dormant seeds imbibed in water for 1 day with 12 h of light with (**e**) detail of the shoot apex and (**f**) detail of the root apex. **g** and **h** Primary dormant seeds imbibed in water for 14 days (12-h photoperiod) with (**i**) detail of the shoot apex and (**j**) detail of the root apex. DAPI (in red, false color) and Alexa 488, immunostaining of H4Ac (in green). Scale bars of 180 and 30 µm

In mature dry seeds (Fig. 5a), H4 acetylation was detected in moderate levels in the vascular tissue nuclei of the radicle, excluding the RAM (Fig. 5c), and in the vascular tissue nuclei of the shoot apex, but not in the SAM or true leaf primordia (Fig. 5b). After 1 day imbibed in water with 12 h of light, a large increase in the number and intensity of nuclei marked with H4Ac signal was detected in the whole embryo (Fig. 5d, Supplementary figure SF. 5, Supplementary table ST. 4). The intensity of the immunosignal was the highest in the shoot apex, including SAM and the leaf primordia (Fig. 5e), the first part of the cotyledons and in the lower-middle hypocotyl. Moderate levels were detected in the radicle, including the RAM (Fig. 5f).

In imbibed primary dormant seeds (PDS), the number of nuclei with H4Ac signal decreased with the depth of dormancy. Seeds imbibed for 14 days (12- h photoperiod) had fewer marked nuclei (and of lower intensity) within the whole embryo in comparison with seeds imbibed for 1 day (Supplementary figure SF. 5, Supplementary table ST. 4), with marks disappearing especially in the SAM and leaf primordia (Fig. 5g–i). Nonetheless, the RAM was still acetylated (Fig. 5j).

#### Secondary dormant seeds

##### DNA methylation

**Fig. 6:**
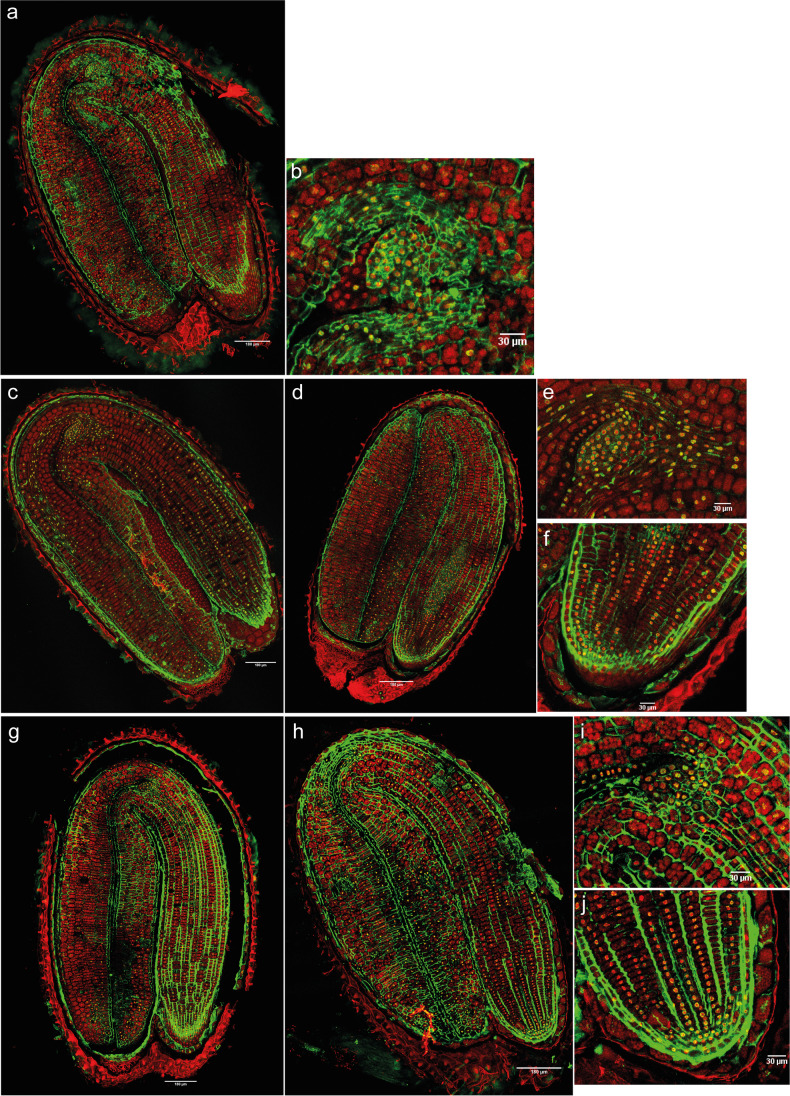
Merged images of immunodetection of 5-mC using confocal microscopy in non-dormant seeds. **a** Non-dormant dry seeds with (**b**) detail of the shoot apex. **c** and **d** Non-dormant seeds imbibed in water for 1 day with 12 h of light with (**e**) detail of the shoot apex and (**f**) detail of the root apex. **g** and **h** Non-dormant seeds imbibed in water for 1 day in darkness with (**i**) detail of the shoot apex and (**j**) detail of the root apex. DAPI (in red, false color) and Alexa 488, immunostaining of 5-mC (in green). Scale bars of 180 and 30 µm

**Fig. 7:**
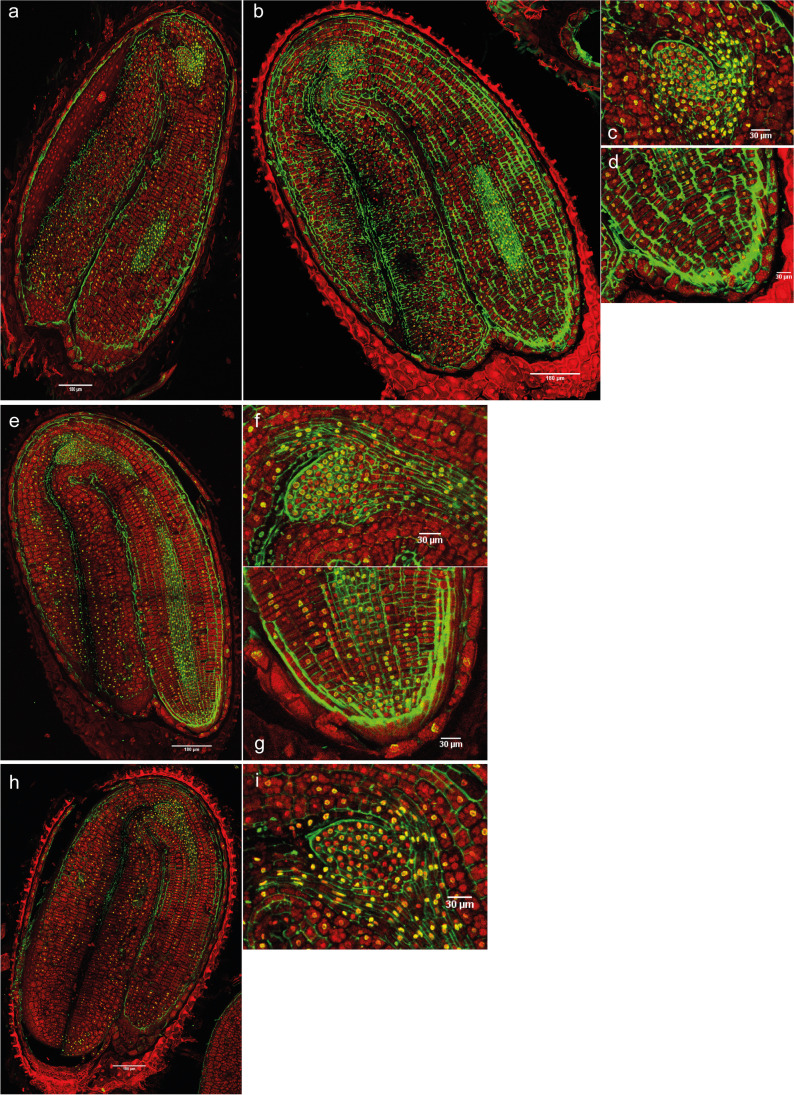
Merged images of immunodetection of 5-mC using confocal microscopy in induced secondary dormant seeds. **a** and **b** Seeds imbibed in water for 3 days in darkness with (**c**) detail of the shoot apex and (**d**) detail of the root apex. **e** Seeds imbibed in water for 7 days in darkness with (**f**) detail of the shoot apex and (**g**) detail of the root apex. **h** Seeds imbibed in water for 14 days in darkness with (**i**) detail of the shoot apex. DAPI (in red, false color) and Alexa 488, immunostaining of 5-mC (in green). Scale bars of 180 and 30 µm

Whilst the DNA methylation signal in non-dormant dry seeds was highest in the leaf primordia (Fig. 6a, b), and became more obvious in the shoot apex, leaf primordia, and vascular tissue nuclei of the meristem after 1 day of imbibition in the light (Fig. 6d), the signal in the whole embryo after 1 day of light was low (Fig. 6c, Supplementary figure SF. 6, Supplementary table ST. 5). By comparison, 1 day in darkness triggered an increase of the 5-mC immunosignal in the SAM, the cotyledons, and in the radicle, with a particularly high immunosignal observed in the nuclei of the RAM in the dark (Fig. 6g–j). By 3 days in the dark, nuclei in the leaf primordia, the SAM and vascular tissue nuclei of the meristem were marked with 5-mC signal (Fig. 7a–c), with these areas having the most intense immunofluorescence within the whole embryo (Fig. 7a–d). In general, the mean intensity per nucleus for the whole embryo was highest at 3 days compared to the other treatments (Supplementary figure SF. 6, Supplementary table ST. 5). At 7 days in darkness, leaf primordia revealed comparably high levels of this modification relative to seeds imbibed for 3 days, whilst the signal in the SAM, vascular tissues of the meristem, radicle, and RAM was reduced (Fig. 7e–g). However, the total intensity of the whole embryo (nuclei × mean intensity) was higher at 7 days than at 3 days (Supplementary table ST. 5). Seeds imbibed in darkness for 14 days showed lower whole embryo fluorescence in comparison with 3 or 7 days (Supplementary figure SF. 6, Supplementary table ST. 5). There were fewer marked nuclei in the leaf primordia, the SAM, and in the cotyledons (Fig. 7h, i), but also in the radicle and RAM. Nevertheless, the SAM presented higher methylation levels than the RAM and radicle.

##### Histone H4 acetylation

**Fig. 8:**
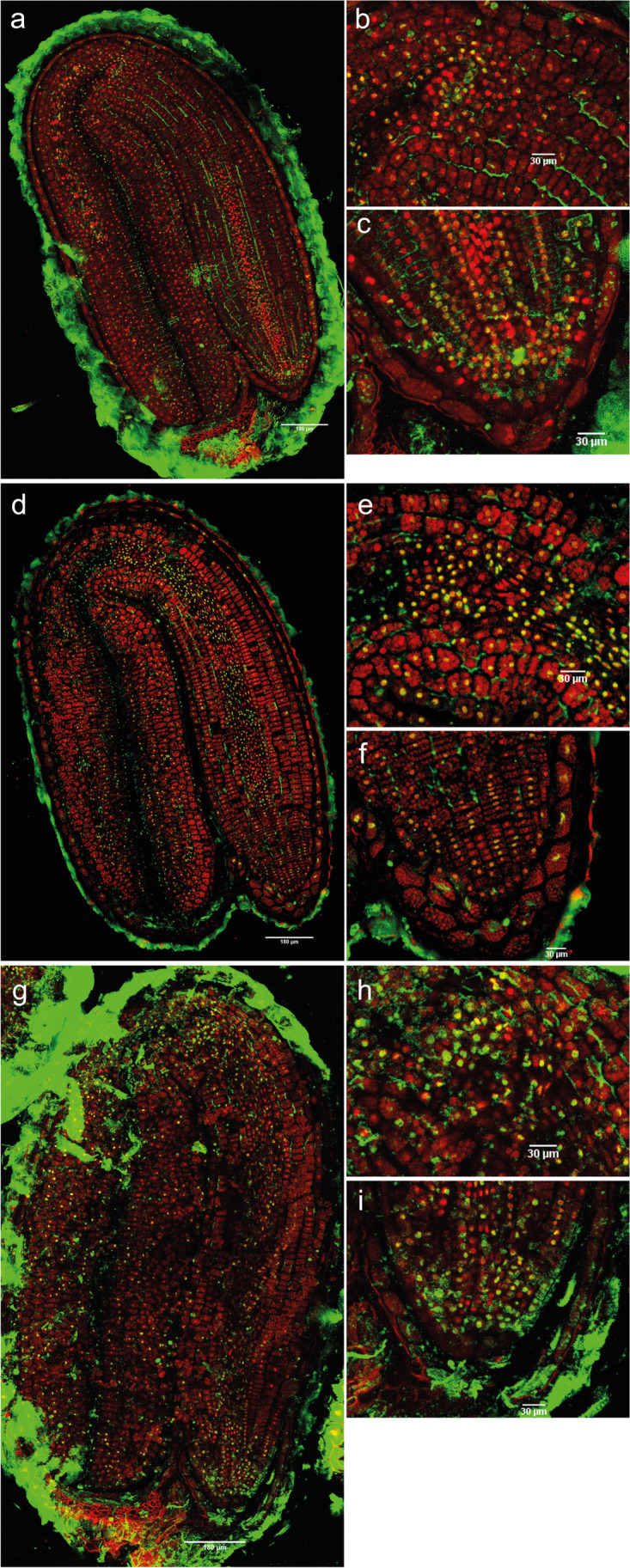
Merged images of immunodetection of H4Ac using confocal microscopy in non-dormant seeds. **a** Non-dormant dry seeds with (**b**) detail of the shoot apex and (**c**) detail of the root apex. **d** Non-dormant seeds imbibed in water for 1 day with 12 h of light with (**e**) detail of the shoot apex and (**f**) detail of the root apex. **g** Non-dormant seeds imbibed in water for 1 day in darkness with (**h**) detail of the shoot apex and (**i**) detail of the root apex. DAPI (in red, false color) and Alexa 488, immunostaining of H4Ac (in green). Scale bars of 180 and 30 µm

H4Ac immunosignal in dry seeds without dormancy (Fig. 8a–c) was detected in the root apex, specifically in the nuclei of the RAM, the cortex, and the pericycle of the radicle (Fig. 8c). When seeds were imbibed for 1 day with 12 h of light, conditions appropriate for germination for non-dormant seeds, there was a large increase in H4-acetylated nuclei (Fig. 8d–f, Supplementary figure SF. 7, Supplementary table ST. 6). The shoot apex presented H4 acetylation in the leaf primordia but not in nuclei of the SAM (Fig. 8e) and the most intense immunofluorescence was localized in the vascular tissue nuclei of the hypocotyl and the elongation zone.

For the induction of secondary dormancy, seeds were imbibed in darkness at the same temperature. In general, whole embryo fluorescence (total and per nucleus) was higher at 1, 3, and 7 days of darkness than in dry seeds or seeds imbibed for 14 days (Supplementary figure SF. 7, Supplementary table ST. 6). After 1 day in darkness, seeds had low levels of H4 acetylation in the vascular tissue nuclei of the hypocotyl and of the elongation zone (Fig. 8g), compared to seeds imbibed in the light. A comparatively high signal was found in the nuclei of the leaf primordia and the RAM (Fig. 8h, i).

**Fig. 9:**
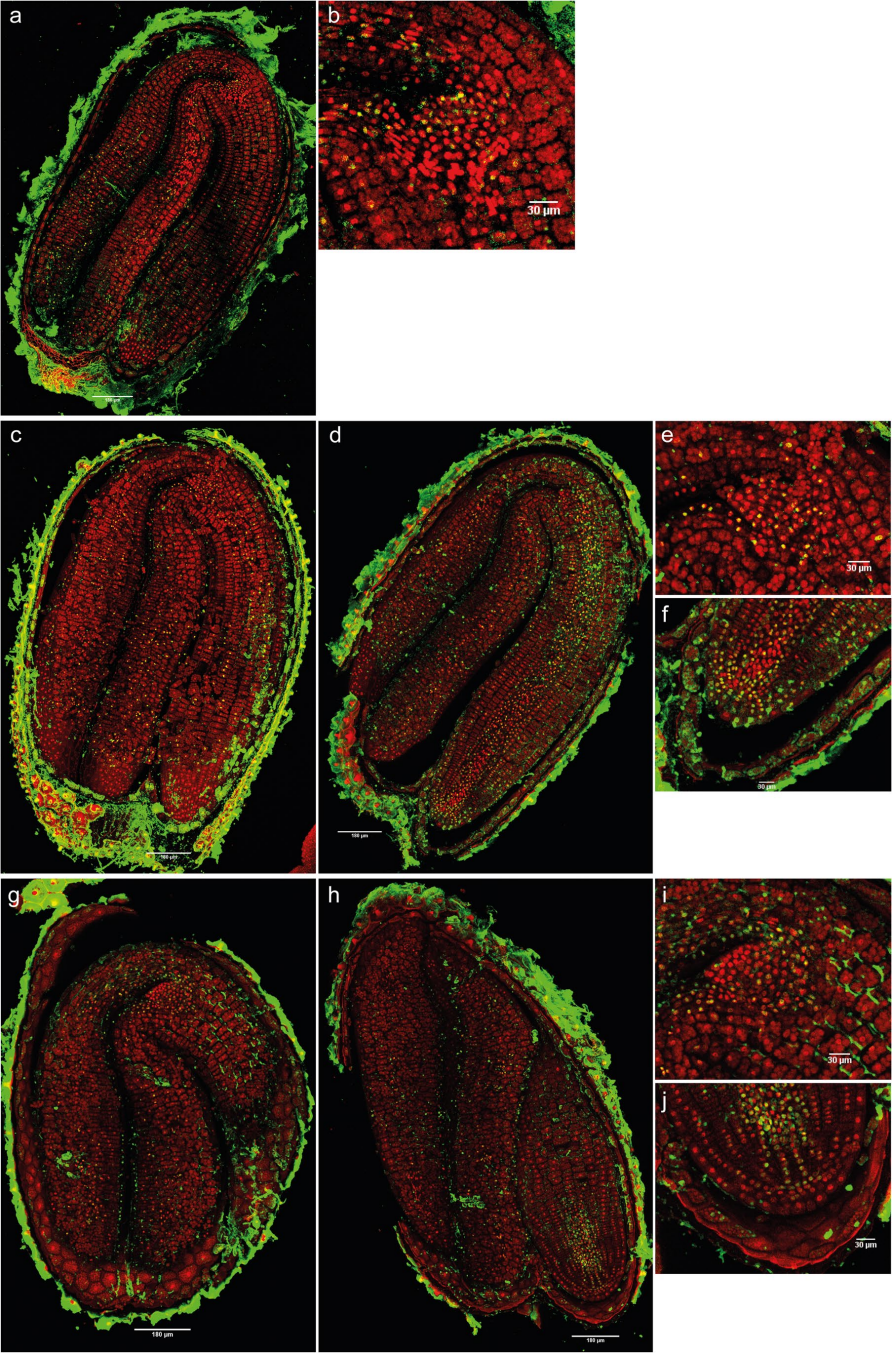
Merged images of immunodetection of H4Ac using confocal microscopy in induced secondary dormant seeds. **a** Seeds imbibed in water for 3 days in darkness with (**b**) detail of the shoot apex. **c** and **d** Seeds imbibed in water for 7 days in darkness with (**e**) detail of the shoot apex and (**f**) detail of the root apex. **g** and **h** Seeds imbibed in water for 14 days in darkness with (**i**) detail of the shoot apex and (**j**) detail of the root apex. DAPI (in red, false color) and Alexa 488, immunostaining of H4Ac (in green). Scale bars of 180 and 30 µm

When seeds were imbibed in darkness for 3 days, H4Ac modification was reduced in the SAM, the leaf primordia, the hypocotyl, and the elongation zone, whilst H4 acetylation levels were maintained in the cotyledons (Fig. 9a, b), compared to seeds imbibed in darkness for 1 day. After 7 days (Fig. 9c, d), very low or no signal was observed in the leaf primordia and the SAM (Fig. 9e), whereas highly intense immunosignal was present in the upper and middle hypocotyl and in the RAM (Fig. 9f). Cotyledons had a weak signal.

Very weak or no immunosignal was observed in the leaf primordia, the SAM, or cotyledons of seeds imbibed in darkness for 14 days (Fig. 9g–i). Nonetheless, immunofluorescence was visualized in the RAM, although not in the epidermis tissue nuclei, like it was in seeds imbibed for 7 days. Overall, the number of nuclei with fluorescence in the whole embryo was lowest at 14 days (Supplementary table ST. 6).

## Discussion

The role of epigenetic regulation in the control of seed dormancy has remained elusive and has only begun to be elucidated in recent years. No other investigation has previously quantified the levels of global DNA methylation of whole seeds in different physiological states of their dormancy cycle. In addition, previous immunohistochemical investigations have used non-dormant dry seeds, germinating seeds, or embryos (Zluvova et al. 2001; Wolny et al. 2014, 2017), but never dormant. The main aim of the present work was to verify the dynamic roles of 5-mC and acetylation of H4 in the regulation of primary and secondary seed dormancy and to detect the levels of these epigenetic marks in specific organs and tissues throughout the process of dormancy induction and maintenance. The use of whole dormant seeds without removal of the testae was an approach methodologically demanding. Dormant seeds showed more obstacles to the passing of antibodies through cell walls than non-dormant or germinating seeds. Washing steps were increased with respect to other protocols, however, that did not lead to an improvement in the elimination of artifacts. Longer incubation times in solutions with enzymes to digest the cell walls than in previous investigations were done. As a result, secondary antibodies, especially in 5-mC immunolocalizations, were attached to the cell walls. In future studies, the analysis of only embryos instead of whole seeds would probably generate images with fewer artifacts. At the same time, using a monoclonal H4 acetylation antibody could possibly prevent these undesired non-specific signals.

### Capsella bursa-pastoris embryo anatomy

The SEM images of shepherd’s purse mature embryos showed that the shoot apical meristem is flanked by two leaf primordia. This feature has been described for the close species *A. thaliana* (Irish and Sussex 1992), but it had not been described for the *Capsella* genus before. However, in *Arabidopsis* seeds, the first two primordia appeared after 48 h of imbibition, whilst in shepherd’s purse, they were already observed in the dry state. These leaf primordia can also be clearly observed in the immunolocalization images (e.g., Fig. 4a, b).

### Overall dynamics of epigenetic marks in (secondary) seed dormancy

In our initial hypothesis for methylation and acetylation dynamics, we proposed an increase of global DNA methylation levels in relation to the depth of dormancy over time and a decrease of H4 acetylation levels during this process. In this way, seeds imbibed for a long period in darkness, such as 14 days in our study, would present higher 5-mC levels and lower H4 acetylation levels than seeds imbibed for a short period, such as 1 day.

Although for prolonged imbibed primary dormant seeds (after shedding from the mother plant and in the soil seed bank), the results obtained were in accordance with the initial hypothesis; this was not the case for secondary dormant seeds. Primary dormant seeds imbibed for 14 days had global DNA methylation levels higher than those imbibed for only 1 day. Nevertheless, secondary dormant seeds after 14 days imbibed in darkness had lower global DNA methylation levels than seeds imbibed for a shorter period, such as 3 days. This could implicate differences in the regulation of primary and secondary dormancy, which would be in accordance with the distinct environmental treatments necessary for triggering germination in primary or secondary dormant seeds.

The immunolocalizations showed that the general patterns of H4Ac and 5-mC in whole seeds were spatially complex. A wide variation of H4Ac and 5-mC was found within and between different tissues of the embryos and between different dormancy states. The dynamics of H4 acetylation agreed with our initial hypothesis, in that we detected a lower number of H4Ac marked nuclei (and of lower intensity) in deeper dormancy conditions. In the case of DNA methylation, primary dormant seeds in a deeper dormancy state had an increased DNA methylation signal but only in specific parts of the seed. On the other hand, during the induction of secondary dormancy, seeds did not have a higher number of nuclei marked or higher intensity of the 5-mC signal as dormancy deepened. However, secondary dormant seeds showed temporal patterns of methylation similar to those found in the global methylation quantification analysis (Fig. 2, Supplementary figure SF. 6).

### 5-mC and H4Ac dynamics in primary dormant seeds

Almost no differences in the quantification of global DNA methylation levels were detected between primary dormant dry seeds and primary dormant seeds imbibed for 1 day with 12 h of light (Fig. 2a, e). A high constant temperature is not adequate for germination of PDS that have not passed through a pre-chilling treatment (primary dormancy is not released) for shepherd’s purse (Popay and Roberts 1970), which could explain the phenomenon.

Primary dormant seeds imbibed in water for 14 days in a 12-h photoperiod had significantly higher global DNA methylation levels than primary dormant seeds imbibed for only 1 day under the same conditions. Future studies could test whether this is also the case for seeds under natural conditions after they have shed from the mother plant and formed part of the soil seed bank. For example, further investigations need to test if these 5-mC levels are maintained, increased, or decreased (as in secondary dormancy induction mentioned below) along with the time of imbibition at a constant temperature. In addition, as imbibition in water at a constant temperature does not mimic natural conditions that occur in the soil seed bank, the effects of diurnal temperature cycling should be explored.

The genomes in dry seeds tend to be under-acetylated (Hodurková and Vyskot 2003). Our analysis of the immunolocalization images for primary dormant dry seeds shows H4 acetylation dynamics that concur with that perspective, and similar to that found in the nuclei of pollen with high chromatin compaction and H4 hypo-acetylation (Janousek et al. 2000).

In contrast, hyper-methylation is known in dry seeds (Hodurková and Vyskot 2003) and global DNA methylation can be high in mature pollen (Janousek et al. 2000). This is not the case for *C. bursa-pastoris* seeds, based on 5-mC immunolocalization. Primary dormant dry seeds had fewer marked nuclei and lower intensity of the signal in general terms (Fig. 4a, Supplementary table ST. 3) in comparison with PDS imbibed for 1 day with 12 h of light (Fig. 4d). These results seem to indicate that DNA methylation levels increase after imbibition of seeds under conditions that do not promote germination. As the DNA methylation signal increased only in specific parts of the seed after 14 days of imbibition, whilst a significant increase in DNA methylation levels was observed in the global quantification, it seems that spatially significant changes in the DNA methylation signal can be both responsible for and potentially masked by the overall differences in the global DNA methylation quantification.

If we focus on the shoot and the root apex, the SAM and the leaf primordia, but not RAM, lost their acetylation signals in the deep primary dormant state (Fig. 5i, j). On the other hand, 5-mC disappeared from the radicle and the RAM, but increased in the SAM under the same conditions (Fig. 4h, i). The shoot apex represents a permanent pluripotent cell line, with self-maintenance of meristematic cells and morphogenesis of the plant body (Zluvova et al. 2001). This could indicate that for the maintenance of a deeper primary dormancy state in seeds, the shoot apex needs to be maintained in a “silenced state” by hyper-methylation, whilst the radicle can present certain levels of histone acetylation. This acetylation of the radicle, including the RAM, may suggest a central role for the radicle/RAM in the response to any ambient changes perceived by the seed and in adjusting the seed dormancy state to them.

### 5-mC and H4Ac dynamics in non-dormant and secondary dormant seeds

Dry seeds of accession -367 that could be induced most rapidly into secondary dormancy had global DNA methylation levels between those of the primary dormant dry seeds—the lowest—and those of the dry seeds of the non-deep dormant accession -799—the highest (Fig. 2a). We could speculate that the higher values presented by the non-deep dormant accession dry seeds in comparison with the deep dormant accession dry seeds might be linked to a relationship between the 5-mC levels and the potential of germination. Whilst a drop in global 5-mC levels was associated with a decrease in the viability of *Quercus robur* L. recalcitrant seeds (Michalak et al. 2015), the two *Capsella* accessions analyzed here (-367 and -799), both retained viabilities near 100% (Supplementary figure SF. 3). Thus, any changes in methylation more likely reflect underlying differences in dormancy rather than seed quality.

When *C. bursa-pastoris* seeds were sown under conditions that promote germination in non-dormant seeds (i.e., 1 day, 30 °C, light), a pronounced decrease in DNA methylation levels was detected in both (-367 and -799) accessions (Fig. 2d, e). This demethylation event accompanies the germination of seeds from many species (Portis et al. 2004; Guangyuan et al. 2006; Meng et al. 2012; Lin et al. 2017; Narsai et al. 2017). Generally, it is known that *A. thaliana* seeds have increased methylation from differentiation to dormancy and decreased methylation post-germination and into the seedling stages (Lin et al. 2017).

With respect to secondary dormancy induction, the DNA methylation signal after 3 days of dark imbibition was high in the SAM, the leaf primordia, and in the vascular tissues of the meristem, but reduced thereafter. This suggests a critical point in the induction of secondary dormancy. The significance of this pattern requires assessment at a range of ecologically meaningful temperatures and conditions of intermittent hydration to mimic realistic field conditions. The cellular 5-mC immunolocalization and global DNA methylation quantification results show very similar patterns (Fig. 2, Supplementary figure SF. 6). After 3 days in darkness, deep and non-deep dormant seeds presented the highest levels of global DNA methylation, showing decreasing levels with longer periods of imbibition time. The immunolocalizations showed a similar pattern, where the highest mean intensity per nucleus in the whole embryo was detected after 3 days in darkness, compared to 7 or 14 days. However, total intensity (nuclei × mean intensity) was highest at 7 days, suggesting a peak in methylation around 3–7 days of darkness.

Comparing the H4Ac signals between the shoot and the root apex in non-dormant dry seeds, the shoot apical meristem did not present any signal opposite to the root apex, where the RAM was marked (Fig. 8a, c). A high increase of H4 acetylated marked nuclei was detected in the whole embryo in germinating conditions, particularly in the hypocotyl and the elongation zone (Fig. 8d, f). This change in the spatial pattern for acetylation may indicate that the chromatin in these areas needs reorganization to enable transcriptional reprogramming, and that these embryo regions are implicated in the induction of germination.

In contrast, after 1 day of imbibition in darkness, which induces secondary dormancy, embryos had lower levels of H4 acetylation in the hypocotyl and the elongation zone (Fig. 8d, g). By 3 days in the dark, H4 acetylation decreased further, except for the cotyledons (Fig. 9a). By 14 days in the dark, the signal had completely disappeared in the SAM, leaf primordia, and the cotyledons (Fig. 9c–e, g–i), whilst the RAM and/or the epidermis tissue nuclei maintained the H4Ac mark (Fig. 9f, j). This is similar to the deeper primary dormant state, where the SAM did not show H4 acetylation signal and the RAM still maintained certain acetylation levels after 14 days of imbibition. This suggests, like for PDS described above, that the radicle could be a center of control that integrates the environmental external signals received by the seed (such as light, temperature, or moisture) during dormancy, thereby indicating the right time for germination.

## Conclusions

In this study, we have linked DNA methylation and histone H4 acetylation patterns to the physiological changes that accompany *C. bursa-pastoris* seed germination and induction and maintenance of primary and secondary dormancy. Our results show that epigenetic marks are dependent on environmental conditions which impact temporal and spatial patterns. For example, global DNA methylation levels increased with primary dormancy depth, whilst levels in secondary dormant seeds peaked at 3–7 days and then declined to lower levels at 14 days of imbibition in darkness.

The immunolocalization results showed that deeply primary dormant seeds had a higher number of 5-mC marked nuclei only in specific parts of the seed, whilst secondary dormant seeds showed temporal patterns of 5-mC similar to those found in the global methylation analysis. In addition, we observed fewer H4Ac marked nuclei (and of lower intensity) in deeper dormant states, for both types of dormancy.

For primary dormant seeds, the 5-mC signal after 14 days of imbibition disappeared in the hypocotyl, elongation zone, and radicle, but it increased in SAM. The SAM lost its acetylation signal after the same period of imbibition, whilst the RAM still presented marked nuclei. In the induction of secondary dormancy, the SAM and RAM showed fewer 5-mC marked nuclei after 7 and 14 days in darkness in comparison with 3 days. However, the SAM presented higher levels of DNA methylation than the RAM. Analyzing the acetylation signal, the SAM had fewer H4 acetylated marked nuclei after 7 and 14 days in darkness in comparison with 3 days, whilst the RAM still presented H4Ac signal. These results seem to indicate that for a deeper primary or secondary dormancy state, the shoot apex needs to be more methylated than the RAM, whilst the latter can maintain certain histone acetylation levels. Keeping the radicle in a (partially) active epigenetic state may be necessary for a proper control of maintenance and release of primary and secondary dormancy.

## Supplementary Information

ESM 1 (PDF 675 kb)

## Abbreviations

SEM: Scanning electron microscopy
5-mC: 5-Methylcytosine
H4Ac: Histone H4 acetylated ();
RAM: Root apical meristem
SAM: Shoot apical meristem
DAPI: 4′,6-Diamidino-2-phenylindole
PDS: Primary dormant seeds
DDA 367: Deep dormant accession -367
NDDA 799: Non-deep dormant accession -799
DD: Days in darkness
