# Supplementary material for: Global DNA methylation and cellular 5-methylcytosine and H4 acetylated patterns in primary and secondary dormant seeds of Capsella bursa-pastoris (L.) Medik. (shepherd’s purse)
Source: Protoplasma. 2021 Jul 2;259(3):595–614. doi: 10.1007/s00709-021-01678-2 (PMC9010400; doi:10.1007/s00709-021-01678-2)

Title

Global DNA methylation and cellular 5-methylcytosine and H4 acetylated patterns in primary and secondary dormant seeds of *Capsella bursa-pastoris* (L.) Medik. (shepherd's purse).

Journal

Protoplasma

Authors

Sara Gomez-Cabellos[a,b,*], Peter E. Toorop[a], María Jesús Cañal[b], Pietro P. M. Iannetta[c], Eduardo Fernández-Pascual[b], Hugh W. Pritchard[a], Anne M. Visscher[a]

[a] Department of Comparative Plant and Fungal Biology, Royal Botanic Gardens, Kew, Wakehurst Place, Ardingly, West Sussex, RH17 6TN, United Kingdom.

[b] Departamento de Biología de Organismos y Sistemas, Universidad de Oviedo, C/Catedrático Rodrigo Uría, 33006, Oviedo, Spain.

[c] The James Hutton Institute, Invergowrie, Dundee, DD2 5DA, Scotland, United Kingdom.

[*] To whom correspondence should be addressed. E-mail address: sara.gomez.cabellos@gmail.com.

Supplementary material

Supplementary table ST. 1

DNA samples extracted in this study

Primary Dormant Conditions -773	Secondary Dormant Conditions -367	Secondary Dormant Conditions -799
Dry seeds Pool 1	Dry seeds 367.1	Dry seeds 799.1
Dry seeds Pool 3	Dry seeds 367.2	Dry seeds 799.2
Dry seeds Pool 5	Dry seeds 367.3	Dry seeds 799.4
1 Day 30 °C 12h light Pool 1	1 Day 30 °C 12h light 367.1	1 Day 30 °C 12h light 799.1
1 Day 30 °C 12h light Pool 3	1 Day 30 °C 12h light 367.2	1 Day 30 °C 12h light 799.2
1 Day 30 °C 12h light Pool 5	1 Day 30 °C 12h light 367.3	1 Day 30 °C 12h light 799.4
14 Days 30 °C 12h light Pool 1	1 Day Dark 367.1	1 Day Dark 799.1
14 Days 30 °C 12h light Pool 3	1 Day Dark 367.2	1 Day Dark 799.2
14 Days 30°C 12h light Pool 5	1 Day Dark 367.3	1 Day Dark 799.4
	3 Days Dark 367.1	3 Days Dark 799.1
	3 Days Dark 367.2	3 Days Dark 799.2
	3 Days Dark 367.3	3 Days Dark 799.4
	7 Days Dark 367.1	
	7 Days Dark 367.2	
	7 Days Dark 367.3	
	14 Days Dark 367.1	
	14 Days Dark 367.2	
	14 Days Dark 367.3	

Supplementary table ST. 2

Differences in the final seed germination proportions of accessions -367 and -799 imbibed in water for all the periods in darkness studied (0, 1, 2, 3 and 7 d) were assessed for significance (*P* < 0.05) by fitting a fully factorial Generalized Linear Model with binomial distribution and logit link (accession or darkness as fixed factors) using R. Asterisks represent significant differences in the interaction of each accession and darkness in the induction of secondary seed dormancy

	X^2	p-value	
**Accession**	89.792	$< 2.2E^{-16}$	***
**Darkness**	291.161	$< 2.2E^{-16}$	***
**Accession:Darkness**	19.454	$1.03E^{-05}$	***

Supplementary figure SF. 1

Formula to calculate the percentage of methylated DNA. The slope is from the standard curve generated with the methylated DNA standards provided in the kit

$$5 - mC\% = \frac{Sample\ OD - NC\ OD}{Slope\ x\ DNA\ ng}\ x\ 100\%$$

Supplementary figure SF. 2

Standard curve obtained in this assay

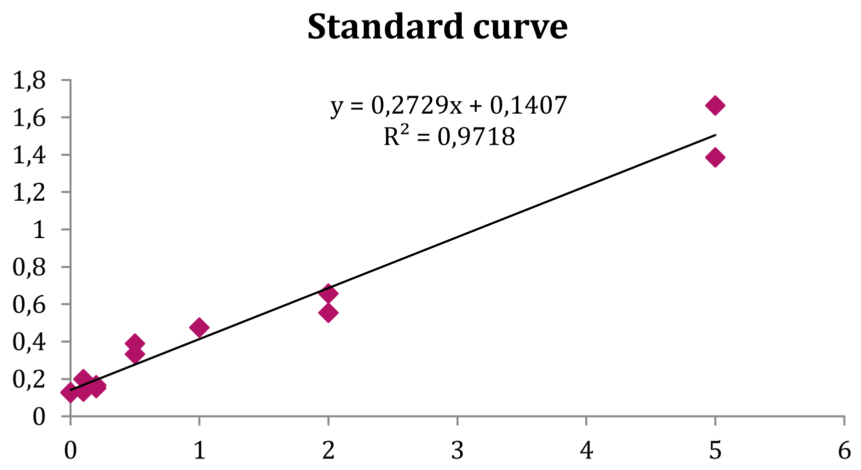

Supplementary figure SF. 3

Mean seed viability of the different accessions among all the treatments studied. Brackets represent the 95 % binomial confidence interval

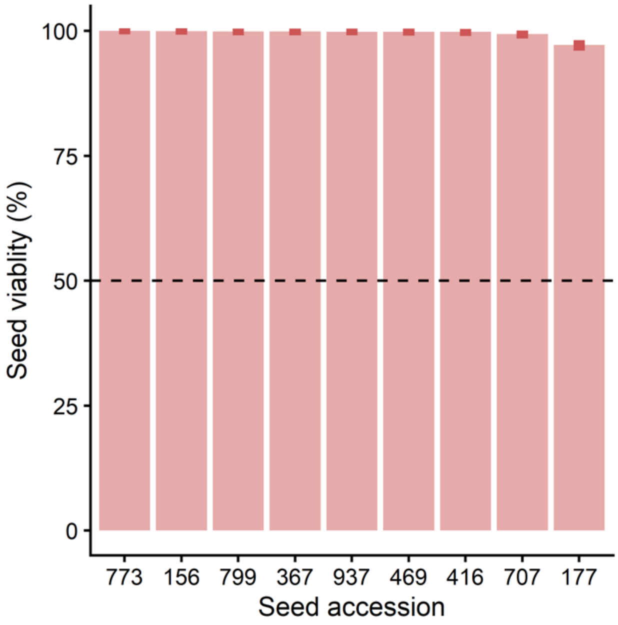

Supplementary figure SF. 4

Quantification of total embryo nuclei 5-mC immunosignal intensity among the different primary dormancy states studied. Data are expressed as mean intensity ± SE. Different letters indicate significant differences after performing one-way ANOVA with post hoc Tukey's HSD with $P = 0.05$

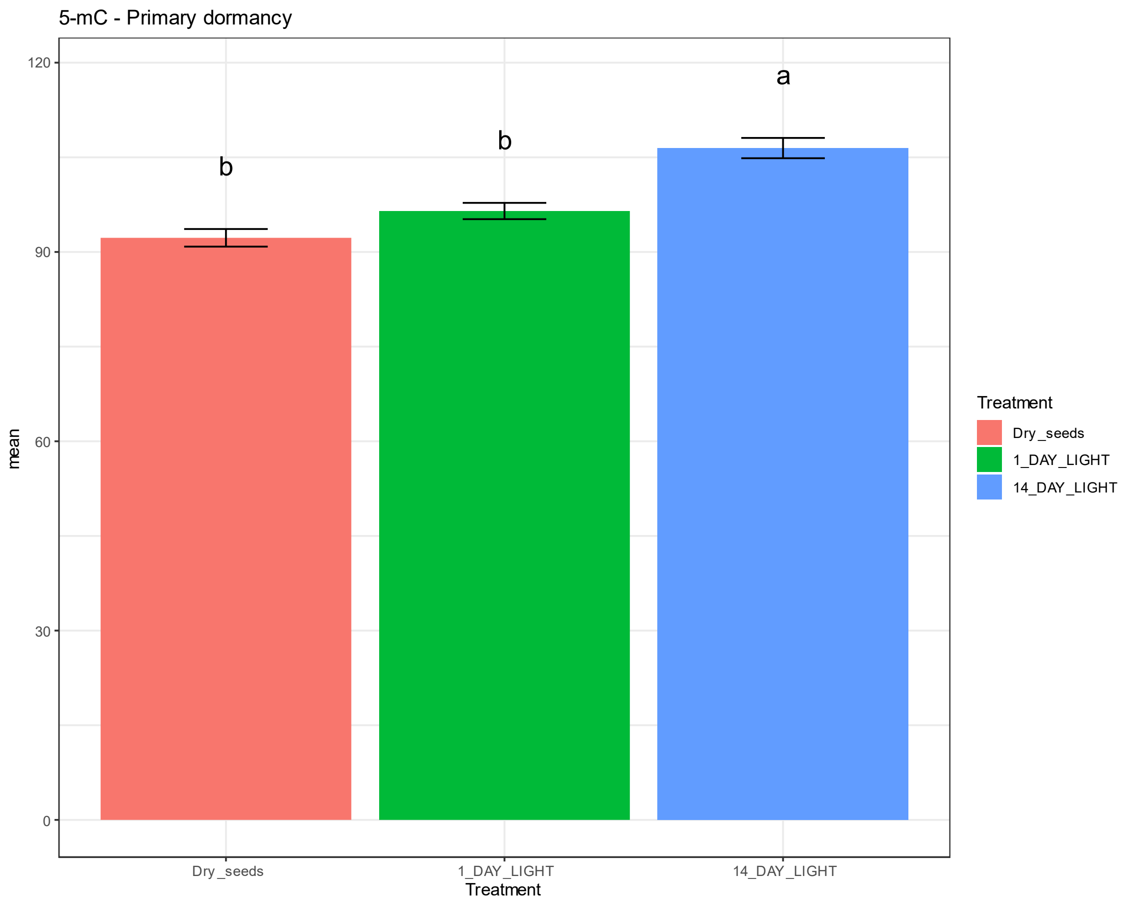

Supplementary table ST. 3

Summary table showing total number of nuclei marked with 5-mC and their mean total intensity for whole embryos among the different primary dormancy states studied. n represents number of nuclei detected and estimation of total intensity is calculated as n × mean

Epigenetic modification	Treatment	Type of dormancy	n	Mean intensity	SD	SE	Estimation of total intensity
5-methylcytosine	Dry_seeds	Primary dormancy	912	92.24	42.13	1.39	84122.88
5-methylcytosine	1_DAY_LIGHT	Primary dormancy	991	96.49	40.61	1.29	95621.59
5-methylcytosine	14_DAY_LIGHT	Primary dormancy	951	106.46	49.57	1.60	101243.46

Supplementary figure SF. 5

Quantification of total embryo nuclei H4-Acetylation immunosignal intensity among the different primary dormancy states studied. Data are expressed as mean intensity ± SE. Different letters indicate significant differences after performing one-way ANOVA with post hoc Tukey's HSD with *P* = 0.05

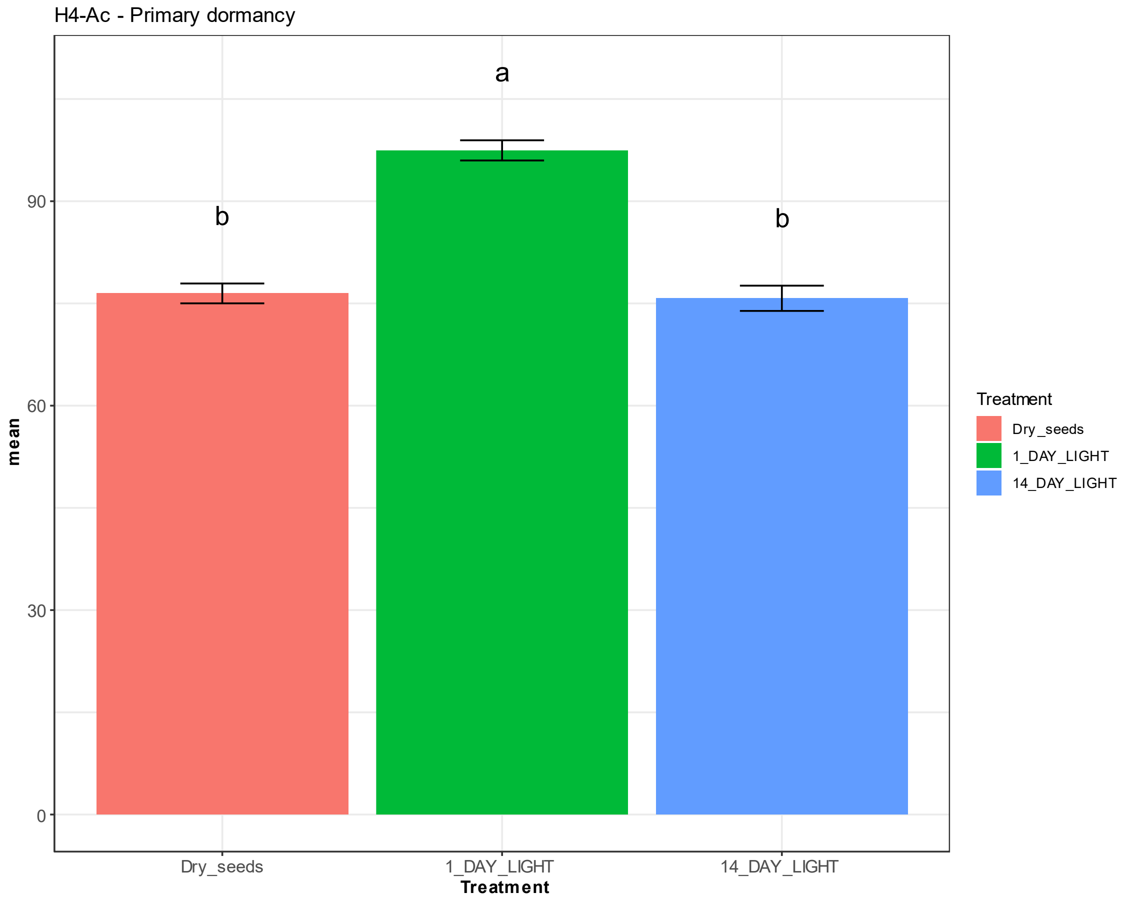

Supplementary table ST. 4

Summary table showing total number of nuclei presenting H4-Acetylation and their mean total intensity for whole embryos among the different primary dormancy states studied. n represents number of nuclei and estimation of total intensity is calculated as n × mean

Epigenetic modification	Treatment	Type of dormancy	n	Mean intensity	SD	SE	Estimation of total intensity
H4-Acetylation	Dry_seeds	Primary dormancy	422	76.47	29.93	1.45	32270.34
H4-Acetylation	1_DAY_LIGHT	Primary dormancy	1074	97.46	48.54	1.48	104672.04
H4-Acetylation	14_DAY_LIGHT	Primary dormancy	480	75.75	40.59	1.85	36360.00

Supplementary figure SF. 6

Quantification of total embryo nuclei 5-mC immunosignal intensity for whole non-dormant embryos and whole embryos in the different secondary dormancy states studied. Data are expressed as mean intensity ± SE. Different letters indicate significant differences after performing one-way ANOVA with post hoc Tukey's HSD with *P* = 0.05

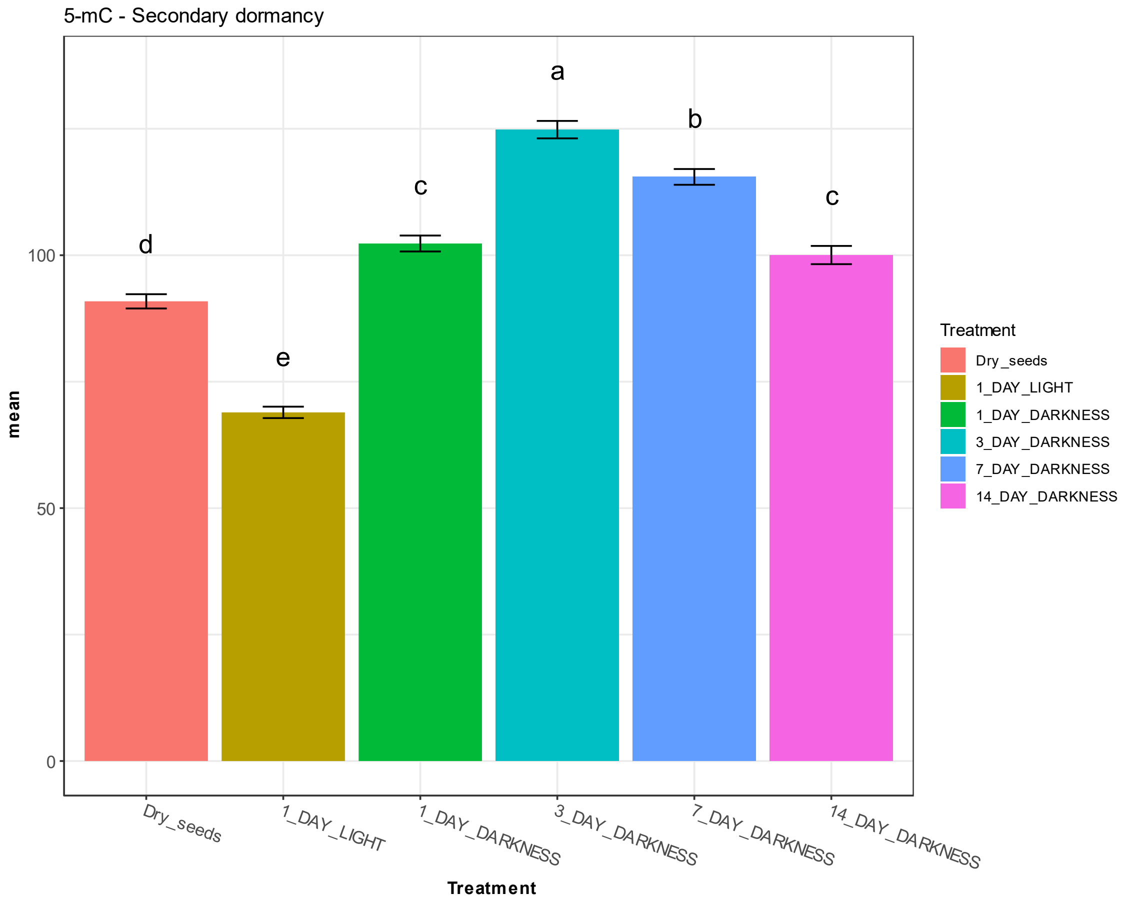

Supplementary table ST. 5

Summary table showing total number of nuclei marked with 5-mC and their mean total intensity for whole non-dormant embryos and whole embryos in the different secondary dormancy states studied. n represents number of nuclei and estimation of total intensity is calculated as n × mean

Epigenetic modification	Treatment	Type of dormancy	n	Mean intensity	SD	SE	Estimation of total intensity
5-methylcytosine	Dry_seeds	Non-dormant	663	90.88	36.37	1.41	60255.89
5-methylcytosine	1_DAY_LIGHT	Non-dormant	753	68.93	31.01	1.13	51896.76
5-methylcytosine	1_DAY_DARKNESS	Secondary dormancy	786	102.32	44.18	1.58	80421.78
5-methyltcytosine	3_DAY_DARKNESS	Secondary dormancy	814	124.83	49.18	1.72	101608.99
5-methylcytosine	7_DAY_DARKNESS	Secondary dormancy	1092	115.48	51.54	1.56	126108.73
5-methylcytosine	14_DAY_DARKNESS	Secondary dormancy	844	100.03	52.37	1.80	84429.87

Supplementary figure SF. 7

Quantification of total embryo nuclei H4-Acetylation immunosignal intensity for whole non-dormant embryos and whole embryos in the different secondary dormancy states studied. Data are expressed as mean intensity ± SE. Different letters indicate significant differences after performing one-way ANOVA with post hoc Tukey's HSD with *P* = 0.05.

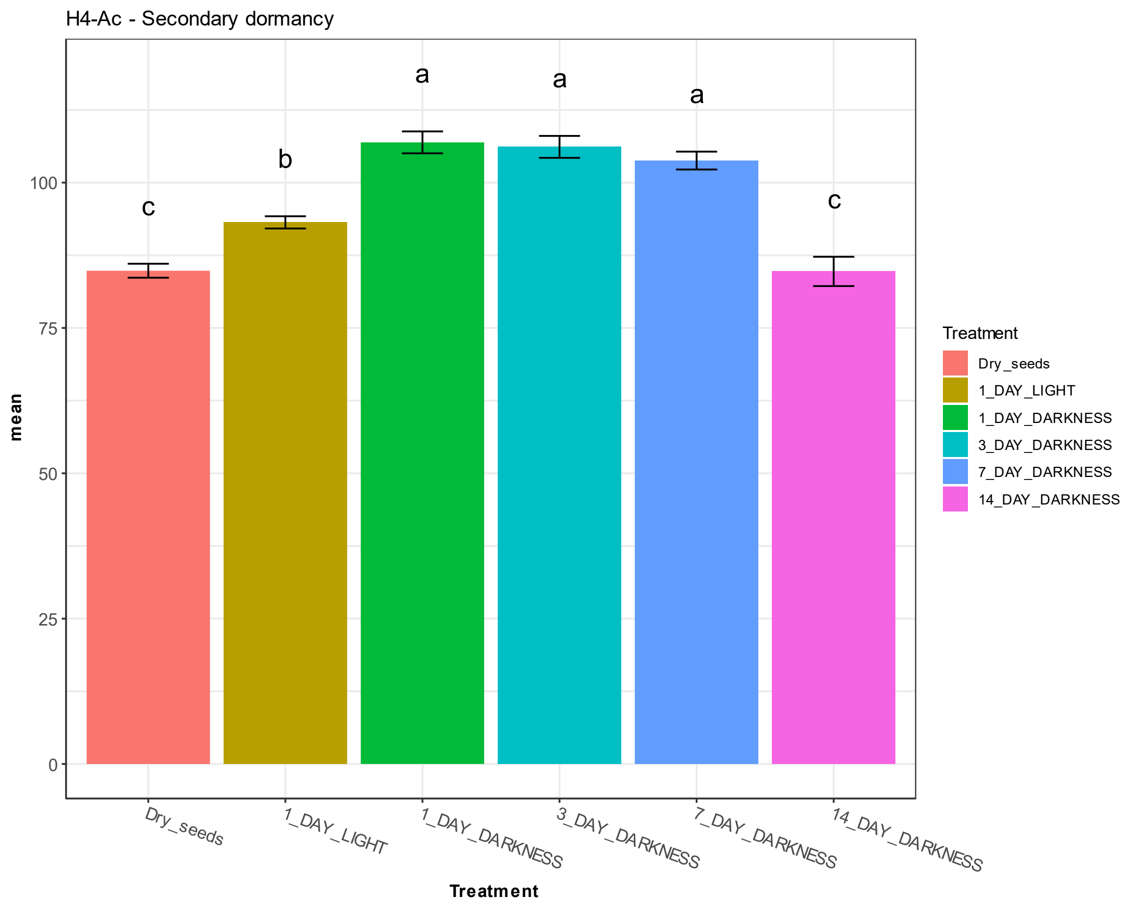

Supplementary table ST. 6

Summary table showing total number of nuclei presenting H4-Acetylation and their mean total intensity for whole non-dormant embryos and whole embryos among the different secondary dormancy states studied. n represents number of nuclei and estimation of total intensity is calculated as n × mean

Epigenetic modification	Treatment	Type of dormancy	n	Mean intensity	SD	SE	Estimation of total intensity
H4-Acetylation	Dry_seeds	Non-dormant	714	84.84	32.13	1.20	60577.53
H4-Acetylation	1_DAY_LIGHT	Non-dormant	1342	93.16	38.57	1.05	125021.75
H4-Acetylation	1_DAY_DARKNESS	Secondary dormancy	716	106.92	50.38	1.88	76558.50
H4-Acetylation	3_DAY_DARKNESS	Secondary dormancy	584	106.15	45.58	1.89	61990.48
H4-Acetylation	7_DAY_DARKNESS	Secondary dormancy	799	103.79	43.43	1.54	82930.97
H4-Acetylation	14_DAY_DARKNESS	Secondary dormancy	271	84.72	41.47	2.52	22960.22

Supplementary figure SF. 8

Cross-section of a *Capsella bursa-pastoris* seed, with the different organs and tissues indicated

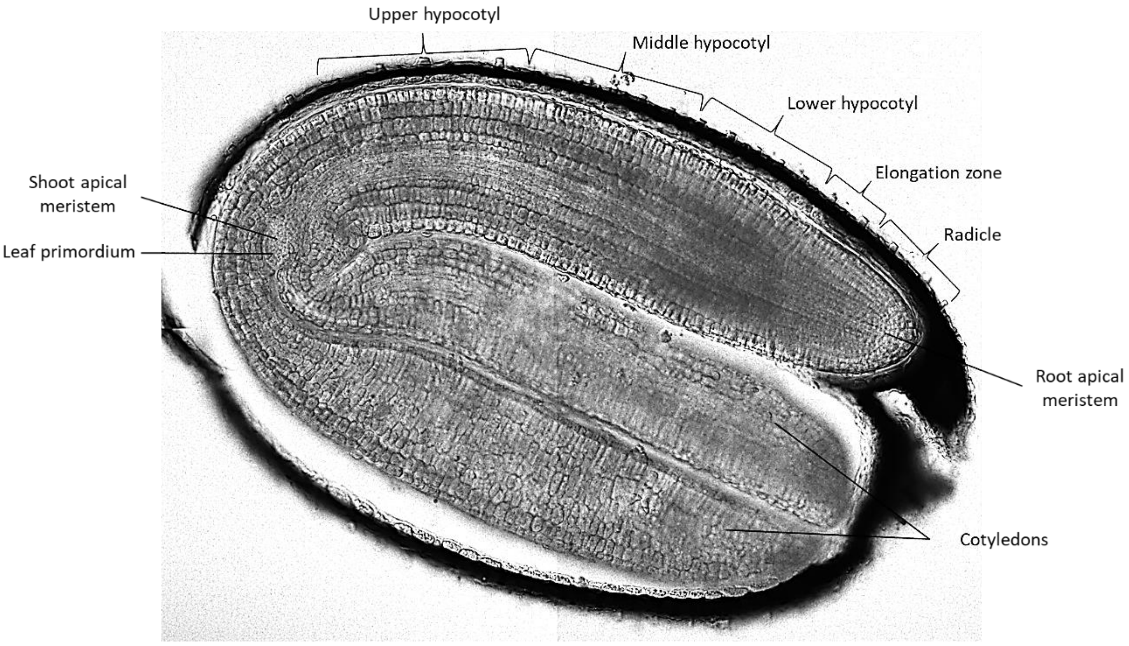

Supplement: Supplementary file 1 — (PDF 675 kb) [file 709_2021_1678_MOESM1_ESM.pdf]
